# Prevalence of vegan/vegetarian diet and eating behavior among Saudi adults and its correlation with body mass index: A cross-sectional study

**DOI:** 10.3389/fnut.2022.966629

**Published:** 2022-09-15

**Authors:** Mohammed AL-Mohaithef

**Affiliations:** Department of Public Health, College of Health Sciences, Saudi Electronic University, Riyadh, Saudi Arabia

**Keywords:** BMI, demographics, physical activity, questionnaire, survey

## Abstract

**Background:**

Globalization has steered the spread of vegetarianism around the world. Vegetarianism has achieved increased acceptance by different populations.

**Objective:**

The present study aims to assess vegetarian diet, and eating behavior prevalence among Saudi adults and their association with demographics and body mass index.

**Method:**

A cross-sectional study conducted on 1,143 Saudi adults [418 (36.6%) males and 725 (63.4%) females]. An online survey questionnaire containing questions on demographics, type of diet, eating behavior and physical activity was provided to participants for self-administration. Statistical analysis was performed to associate demographic and eating behavior variables with the type of diet using Pearson's Chi-square test and Spearman's partial correlation test was used to correlate BMI and eating behavior.

**Results:**

Prevalence of veganism was 4.7% (*n* = 54/1,143) and vegetarianism was 7.8% (89/1,143). A significantly higher prevalence of vegan diet was observed in females than males (79.6% vs. 20.4%, *p* < 0.0001). A significantly higher proportion of participants on vegetarian diet selected “Always” as response for eating breakfast, vegetables and fruits as well as for eating or drinking dairy foods, and for eating canned food than participants on non-vegetarian diet (*p* < 0.0001). A significantly higher proportion of participants on vegan diet selected ‘Never' for eating fast-food and fried food as well as for drinking fizzy or soft drinks (*p* < 0.0001). A positive moderate correlation was found between BMI and eating fast-food and fried food [r_(1, 140)_ = 0.529, *p* < 0.0001], drinking fizzy or soft drinks with meals [r_(1, 140)_ = 0.495, *p* = 0.001], and eating canned food [r_(1, 140)_ = 0.510, *p* < 0.0001].

**Conclusion:**

Our study shows that vegan and vegetarian diet have gained access into the lifestyle of Saudi adults with a prevalence of 4.7 and 7.8%, respectively. Participants on vegetarian diet showed better lifestyle like higher physical activity level, higher consumption of fruits, vegetables, dairy products and low intake of fast-foods and fizzy beverages.

## Introduction

The Global Burden of Diseases report (2015) highlighted an increased prevalence of obesity in Eastern Mediterranean Region (EMR) 21%, much higher than the global obesity average 12% (1). A number of studies were conducted in EMR during 2016-2017 and reported lowest obesity rates 17.0% in Yemen and highest 32.3% in United Arab Emirates (2, 3). A nation-wide survey 2020 from Saudi Arabia reported the weighted prevalence of obesity as 24.7% and unweighted prevalence 21.7% (4). The main reason of high obesity rates in EMR includes nutrition transition, inactivity, and urbanization (5).

Vegetarian diet has gained popularity in western countries and had shown variation in the prevalence rate from 4.3 to 9% (6–9) among the general population, with the highest prevalence from India (30%) (10). A recent nutrition report from Germany stated that the number of vegetarians and vegans have doubled from pre-COVID (5%) to post-COVID (10%) (11). Vegetarians refer to those who do not eat any meat, poultry or fish and may or may not consume egg or dairy products (12), while vegan refers to those who refrain from eating any animal product including dairy, eggs and other animal-derived food (12).

Scientific research findings reported vegan diet is associated with various health benefits (13–15). Studies conducted in western countries have consistently shown that vegetarian diet is associated with low BMI and lower blood pressure (16–18). Further, a vegetarian diet has presented positive effect on preventing and treating non-communicable diseases (17, 18). Despite the health benefits of vegetarian diet, adopting a vegan/vegetarian diet may lead to micro-nutrient deficiencies and can result in negative consequences like impaired cognition, muscular pain, neural tube defect, reduced physical performance and endurance (19, 20). However studies have reported a well-planned vegan and vegetarian diet is capable to fulfill nutrients needs of an individual and may be beneficial to their health (14, 21).

The major transitional changes in lifestyle usually occur in youths, as parental supervision is reduced and new friend relationships are established. The feeling of independence leads to the establishment of personal principles and choices, including diet and eating habits. The change in principles (ethical, environmental, and psychological) is reflected by the adoption of certain dietary patterns (22).

As the overweight and obesity prevalence are high in Saudi Arabia, vegan and vegetarian diet can help to mitigate the problem, information regarding prevalence of vegetarianism among adult Saudi nationals is crucial. Therefore, this study aims to assess vegetarian diet, and eating behavior prevalence among Saudi adults and their association/correlation with demographics and body mass index.

## Materials and methods

### Study design

The present cross-sectional study was conducted in February-March, 2022 after receiving approval from the Research Ethics Committee, Saudi Electronic University, Saudi Arabia. The study was carried out among Saudi adults from 4 cities of Saudi Arabia (Riyadh, Dammam, Jeddah and Abha). Malls were selected as the place to recruit the participants as it is one of the common visiting places of Saudi nationals during the weekends. Randomly 2–4 malls were selected from each city to visit on weekends (Fridays and Saturdays) between 1 pm to midnight.

### Participants

A total of 1,282 Saudi nationals in the age group 18–45 years, who agreed and gave their informed consent to participate in study, were recruited. All the eligible participants height and weight was measured using a portable stadiometer weighing scale machine (Model HW-700z, LEKA) which was capable of measuring height range (60–200 cm with 0.5 cm minimum division) and weight range (8–200 kg with 0.1 kg minimum division). After the measurements the participants completed the online survey questionnaire. Among 1,282 participants, 139 (10.8%) participants reported to be ignorant about the vegetarian diet, so were excluded. After data cleaning a total 1,143 participants' complete and correct data was available for the analysis.

### Measure

BMI was calculated using World Health Organization (WHO) standard formula (23). Participants were categorized as “Under-weight” for BMI <18.5 kg/m^2^, “Normal weight” for 18.5–24.9 kg/m^2^, “Over-weight” for BMI 25–29.9 kg/m^2^, and “Obese” for BMI 30 kg/m^2^ and above according the WHO classification (23).

### Questionnaire

A closed-ended multiple-choice questionnaire was used to assess the diet type, eating behavior and physical activity level of the participants. The questionnaire had 3 sections; the first section included demographic questions (age, sex, education level, BMI). The second section had questions on the awareness of a vegetarian diet, types of vegetarian diet and additional questions for the vegetarian participants included reason for adapting vegan/vegetarian diet, duration of practicing the diet and favorite substitute for meat. The participants were classified as vegan, vegetarian, and non-vegetarian. Vegan refers to those who refrain from eating any animal product including dairy, eggs and other animal-derived food; vegetarians refers to those who do not eat any meat, poultry or fish; while non-vegetarians refers to those who consume meat, poultry, fish and their products (12). The third section had questions related to the eating behavior of the participants (like eating breakfast daily, having three meals a day, fruit & vegetable and dairy product intake, fast-food consumption, sugar-sweetened beverages consumption). The eating behavior questions are presented in a 5-Likert scale with a score “0” for “Never”, “1” for “Rarely”, “2” for “Sometimes”, “3” for “Often” and “4” for “Always”. The physical activity level of participants was assessed by using International Physical Activity Questionnaire-SF questions (24, 25). The criteria used to classify the physical activity level are shown in Table 1.

**Table 1:** Criteria used to classify physical activity level.

	**Low intensity (Walking)**	**Moderate intensity** **(Cycling, swimming)**	**Vigorous intensity (Gardening, climbing stairs)**
“High” activity Graded “3”	Every day (>60 min)	At least 5 days (>60 min)	At least 3 days (>60 min)
	7 or more days of any combination of walking, moderate intensity or vigorous intensity activities.		
“Moderate” activity Graded ‘2'	Every day (>30 min)	5 or more days (>30 min)	3 or more days (>30 min)
	5 or more days of any combination of walking, moderate intensity or vigorous intensity activities.		
“Low” activity Grade “1”	3–5 days	3–5 days	1–3 days
	<5 days of any combination of walking, moderate intensity or vigorous intensity activities.		
Sedentary Graded “0”	0–2 days	0–2 days	0 days

### Ethical statement

The study was approved by the Saudi Electronic University Research Ethics Committee (SEUREC-−22,008, 13^th^ February 2022).

### Statistical Analysis

IBM SPSS Version 24 (IBM Corp, Armonk, NY, United States) was used for the statistical analysis. The demographic characteristics of the participants and their responses to the type of diet, physical activity and eating behavior are reported as numbers and percentages. The comparison of the demographic characteristics, and eating behavior were performed between the types of diet using Pearson's chi-square test. Spearman's partial correlation analysis was used to correlate BMI with eating behavior by controlling for physical activity. A *p*-value of < 0.05 was considered significant.

## Results

**Figure 1:**
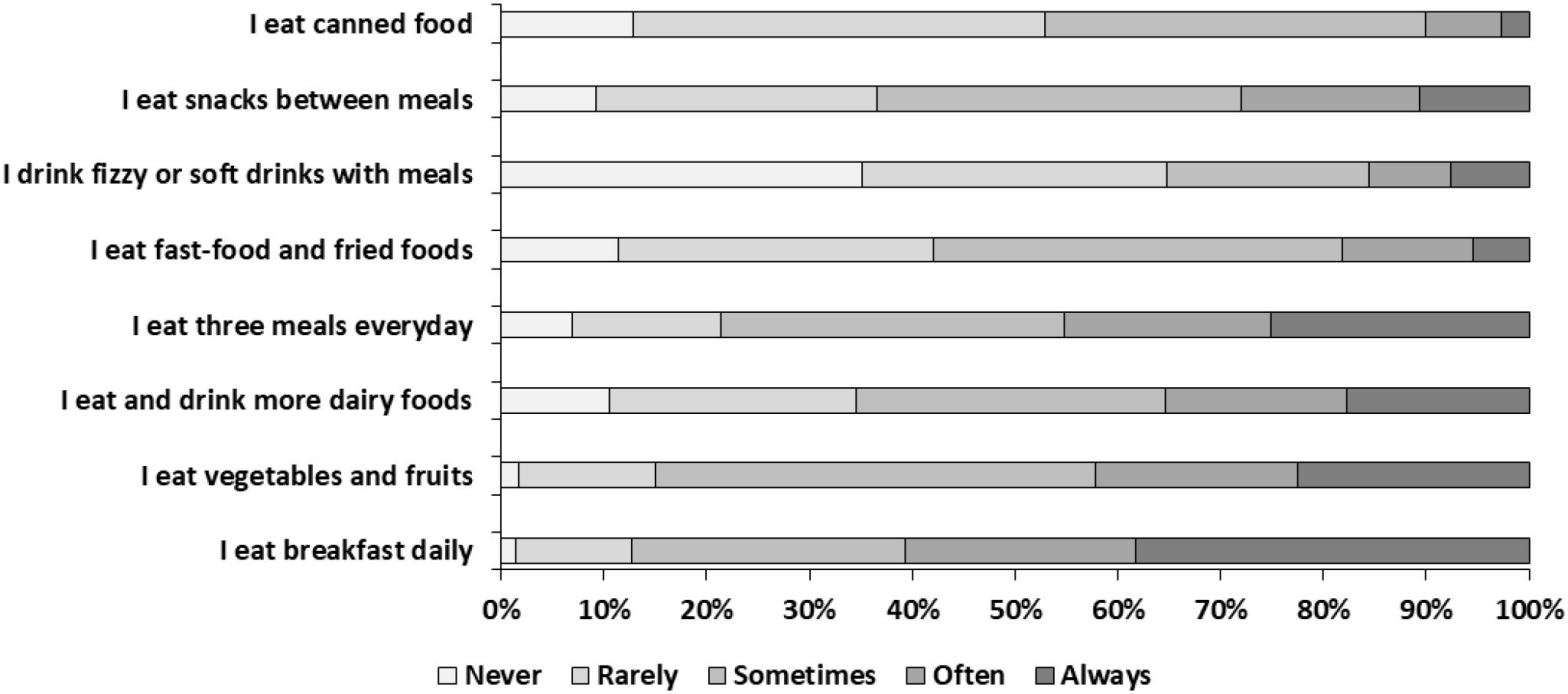
Distribution of eating behavior in the study population.

A total of 1,143 Saudi adults (35.5% males and 64.5% females) were included in the study for statistical analysis. The socio-demographic characteristic of the participants is presented in Table 2. Among the study participants 64.5% (*n* = 737) participants were in the 18–30 age group, and 83.2% (*n* = 951) participants were graduates. The participants recruited from Riyadh were 45.5% (*n* = 520) followed by Dammam 32.5% (*n* = 371). The BMI (mean ± SD) of the participants was 25.5 ± 5.3 kg/m^2^. The classification of BMI into categories revealed that 41.0% (*n* = 469) participants had normal weight, while prevalence of under-weight, over-weight and obesity was 8.8, 31.2, and 19.0%, respectively. The high level of physical activity was reported by 41.3% (*n* = 472) participants. The eating behavior reported by the participants is shown in Figure 1. “Always” was selected as response by 38.2% (*n* = 437) participants for eating breakfast daily, and by 22.2% (*n* = 254) participants for eating three meals a day, while <10% participants selected “always” as a response for eating fast food and fried foods, drinking fizzy or soft drinks with meals as well as eating canned food.

**Table 2:** Socio-demographic characteristics of participants (*n* = 1,143) based on type of diet.

	**Total** ***n* = 1,143**	**Vegan** ***n* = 54**	**Vegetarian** ***n* = 89**	**Non-vegetarian** ***n* = 1,000**	**Statistics**
**Age (years)**					X${}_{\left(2\right)}^{2}$ = 7.15, *p* = 0.028
18–30 31–45	64.5% (737) 35.5% (406)	77.8% (42) 22.2% (12)	71.9% 28.1% (25)	63.1% (631) 36.9% (369)	
**Sex**					X${}_{\left(2\right)}^{2}$ = 8.15, *p* = 0.017
Male Female	36.6% (418) 63.4% (725)	20.4% (11) 79.6% (43)	43.8% (39) 56.2% (50)	36.8% (368) 63.2% (632)	
**Education**					X${}_{\left(4\right)}^{2}$ = 4.96, *p* = 0.29
High school Graduate Postgraduate	13.0% (149) 83.2% (951) 3.8% (43)	13.0% (7) 85.2% (46) 1.9% (1)	15.7% (14) 84.3% (75) 0.0% (0)	12.8% (128) 83.0% (830) 4.2% (42)	
**City**					
Riyadh Dammam Jeddah Abha	45.5% (520) 32.5% (371) 12.3% (141) 9.7% (111)	35.2% (19) 51.9% (28) 5.6% (3) 7.4% (4)	38.2% (34) 37.1% (33) 16.9% (15) 7.9% (7)	46.7% (467) 31.0% (310) 12.3% (123) 10.0% (100)	
**BMI (kg/m** ^**2**^ **)**					X${}_{\left(6\right)}^{2}$ = 77.56, *p* <0.0001
<18.5 18.5–24.9 25–29.9 ≥30	8.8% (100) 41.0% (469) 31.2% (357) 19.0% (217)	25.9% (14) 74.1% (40) 0.0% (0) 0.0% (0)	16.1% (9) 49.7% (31) 47.2% (42) 7.9% (7)	7.7% (77) 39.8% (398) 31.5% (315) 21.0% (210)	
**Physical activity**					X${}_{\left(6\right)}^{2}$ = 123.46, *p* <0.0001
Sedentary Low Moderate High	20.2% (231) 16.3% (186) 22.2% (254) 41.3% (472)	11.1% (6) 7.4% (4) 42.6% (23) 38.9% (21)	1.1% (1) 2.2% (2) 3.4% (3) 93.3% (83)	22.4% (224) 18.0% (180) 22.8% (228) 36.8% (368)	

Data are presented as prevalence (along with number of participants). BMI, body mass index; χ^2^, chi-square. P, p-value for difference between the groups. Non-vegetarian—those who consume fruits, vegetables, pulses or beans, animal products (chicken or meat, fish, eggs, milk or curd) either daily, weekly or occasionally. Vegetarians—those who avoid all flesh foods but consume egg and/or dairy products. Vegan—those who avoid all foods and ingredients from animal sources.

**Figure 2:**
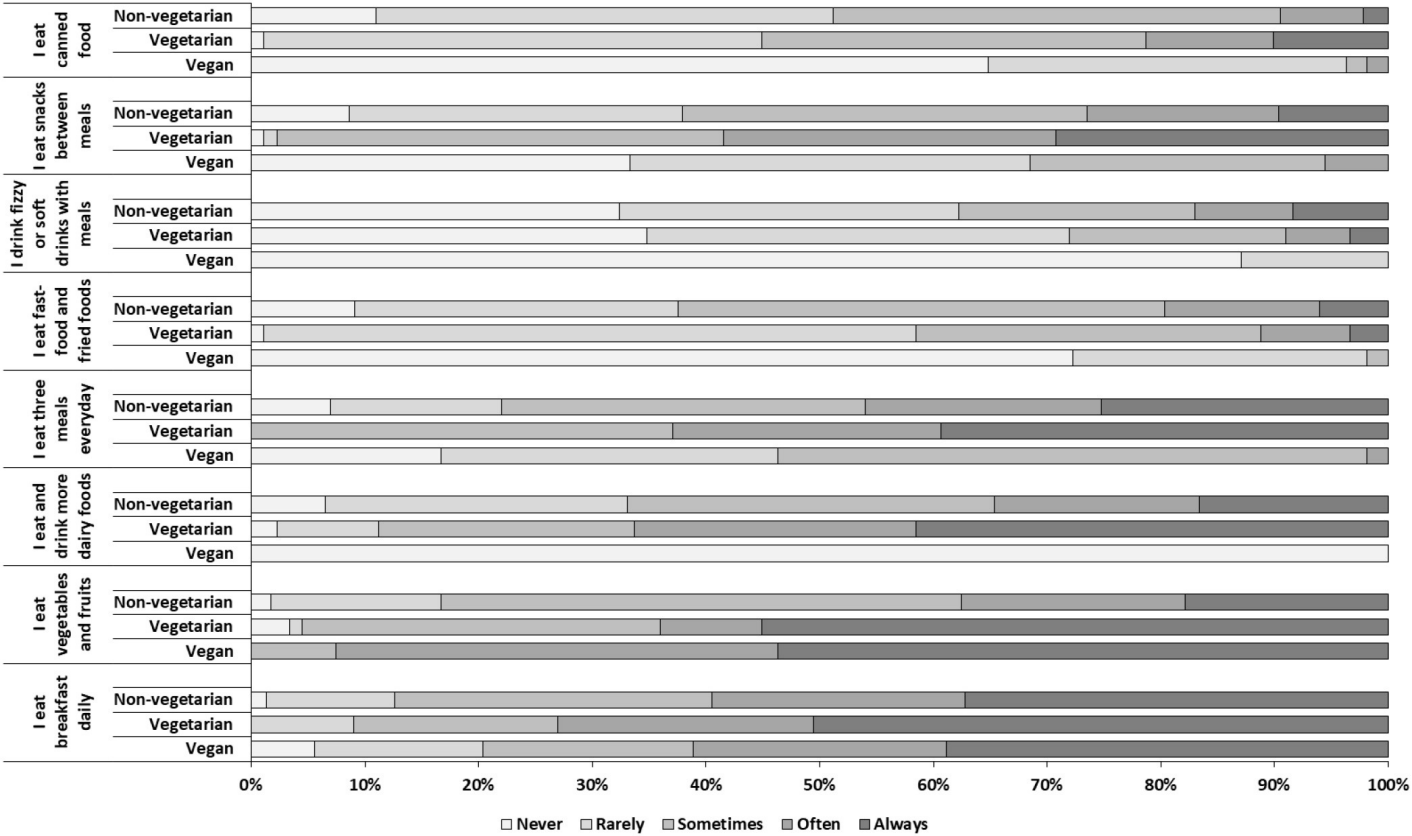
Distribution of eating behavior based on type of diet in the study population.

The prevalence of vegan and vegetarian diet was 4.7% (*n* = 54/1,143) and 7.8% (89/1,143), respectively. The socio-demographic characteristic of the participants based on type of diet is shown in Table 2. A significantly higher prevalence of vegan diet was observed in age group 18–30 years and among females compared to their counter parts (*p* < 0.05). A significant difference was observed for BMI between vegan, vegetarians, and non-vegetarian participants [X${}_{\left(6\right)}^{2}$ = 77.56, *p* < 0.0001], the prevalence of underweight was higher in vegan (25.9%) followed by vegetarian participants (16.1%) than non-vegetarians participants (7.7%) and inversely, obesity prevalence was higher among non-vegetarian participants (21.0%) than vegetarian participants (7.9%). Comparison of physical activity with type of diet showed a significant difference between vegan, vegetarian, and non-vegetarian participants [X${}_{\left(6\right)}^{2}$ = 123.46, *p* < 0.0001] (Table 2). The distribution of eating behavior reported by the participants based on the type of diet is shown in Figure 2. A significantly higher proportion of participants on vegetarian diet selected “Always” as response for eating breakfast daily [X${}_{\left(8\right)}^{2}$ = 17.24, *p* = 0.028], eating vegetables and fruits [X${}_{\left(8\right)}^{2}$ = 132.28, *p* < 0.0001] as well as for eating or drinking dairy foods [X${}_{\left(8\right)}^{2}$ = 524.10, *p* < 0.0001], for eating three meals daily [X${}_{\left(8\right)}^{2}$ = 72.07, *p* < 0.0001], for eating snacks between meals [X${}_{\left(8\right)}^{2}$ = 114.85, *p* < 0.0001] and for eating canned food [X${}_{\left(8\right)}^{2}$ = 171.85, *p* < 0.0001] than participants on non-vegetarian diet (*p* < 0.0001), while significantly higher proportion of participants on vegan diet selected “never” as a response for eating fast food and fried foods [X${}_{\left(8\right)}^{2}$ = 247.11, *p* < 0.0001], and drinking fizzy or soft drinks with meals [X${}_{\left(8\right)}^{2}$ = 73.97, *p* < 0.0001] (Figure 2).

The participants who selected vegan and vegetarian diet were provided with additional questions regarding reasons for adapting vegetarian diet, duration on vegetarian diet and favorite substitute for meat (Table 3). Among the 143 participants who reported to be on vegetarian diet, 37.8% (*n* = 54) were on vegan diet, 25.2% (*n* = 36) were on semi-vegetarian diet and 19.6% (*n* = 28) were on lacto-ova diet. The primary reason for adapting vegan diet was animal ethics (37.0%, *n* = 20), while vegetarian diet adapting mainly for better health/nutrition 59.5% (*n* = 33). 72.9% (*n* = 41) participants had adapted vegan diet within a year of the survey, while 14.6% (*n* = 13) were following a vegetarian diet for more than 10 years. The favorite meat substitute was soya in both groups (Table 3).

**Table 3:** Distribution of reason for adopting vegan/vegetarian diet, duration and favorite meat substitute among vegan and vegetarian participants (*n* = 54).

	**Vegan** ***n* = 54**	**Vegetarian** ***n* = 89**
**What is the primary reason you decided to become vegetarian?**		
For better health/nutrition Weight control Animal ethics I don't like animal products I have a health condition My friends or family are vegetarian Other	18.5% (10) 31.5% (17) 37.0% (20) 13.0% (7) 0.0% (0) 0.0% (0) 0.0% (0)	59.5% (53) 7.9% (7) 3.4% (3) 14.8% (8) 14.8% (8) 1.1% (1) 10.1% (9)
**How long have you been a vegetarian**		
<1 year 1–2 years 2–4 years 4–10 years >10 years	72.9% (41) 11.1% (6) 13.0% (7) 0.0% (0) 0.0% (0)	38.2% (34) 5.6% (5) 24.1% (13) 27.0% (24) 14.6% (13)
**What is your favorite meat substitute**		
Soya Tofu Egg Quorn Other	59.3% (32) 33.3% (18) 0.0% (0) 7.4% (4) 0.0% (0)	23.6% (21) 15.7% (14) 16.8% (15) 1.1% (1) 42.7% (38)

Data are presented as prevalence (along with number of participants).

**Figure 3:**
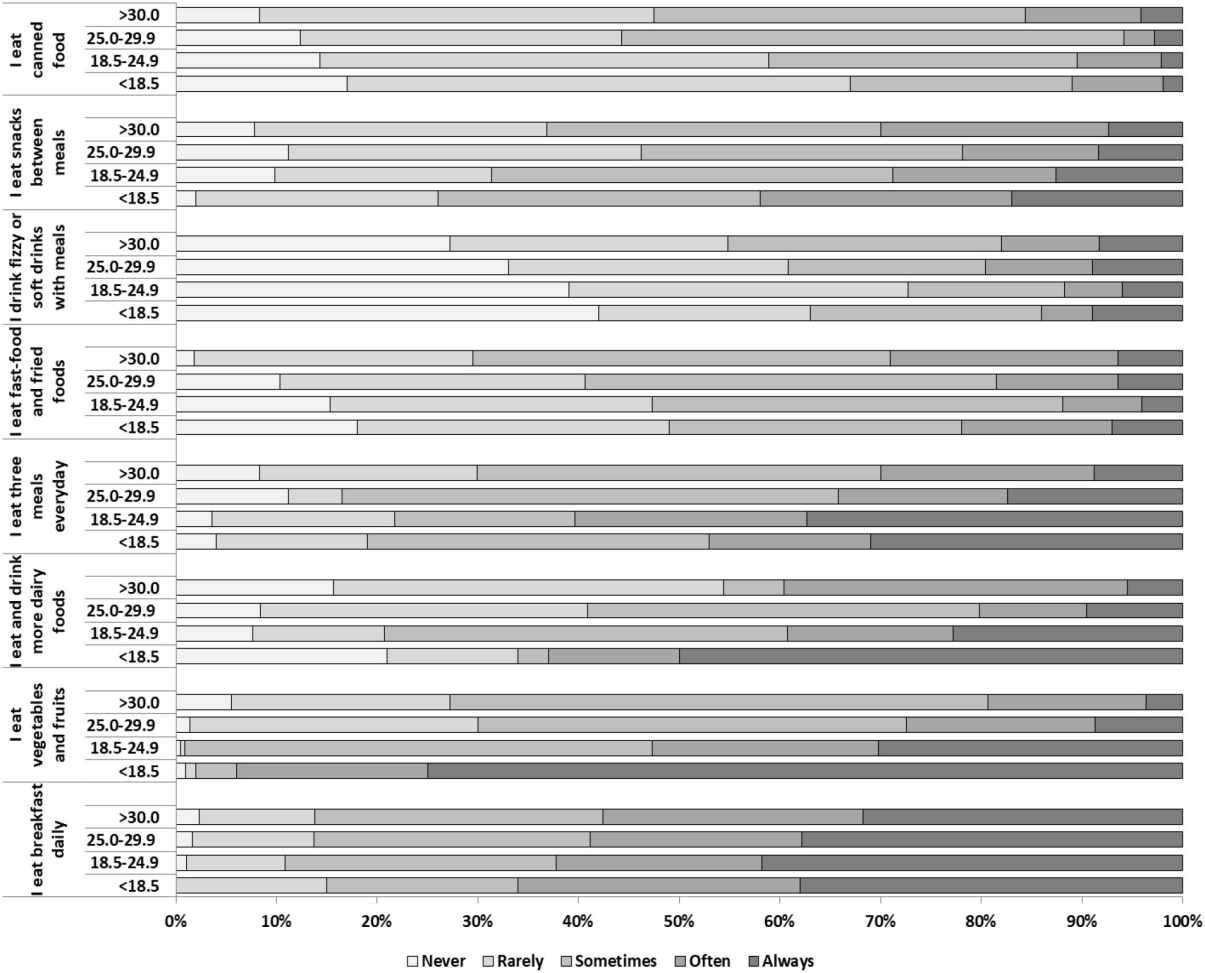
Distribution of eating behavior based on BMI category in the study population..

The Spearman's partial correlation analysis controlling for physical activity showed a negative weak correlation between BMI and eating vegetables and fruits [r_(1, 140)_ = −0.269, *p* < 0.0001], while a positive weak correlation was observed between BMI and eating and/or drinking dairy foods [r_(1, 140)_ = 0.347, *p* < 0.0001], eating three meals a day [r_(1, 140)_ = 0.125, *p* < 0.0001] and eating snacks between meals [r_(1, 140)_ = 0.218, *p* < 0.0001]. A positive moderate correlation was found between BMI and eating fast-food and fried food [r_(1, 140)_ = 0.529, *p* < 0.0001], drinking fizzy or soft drinks with meals [r_(1, 140)_ = 0.495, *p* = 0.001], and eating canned food [r_(1, 140)_ = 0.510, *p* < 0.0001] (Figure 3).

## Discussion

Since there are limited research studies on the prevalence of vegetarianism from Saudi Arabia, the study was designed to assess prevalence of vegetarian diet, eating behavior and to correlate them with demographics and BMI. The present study is the first and only study presenting the prevalence of vegetarianism and eating behavior of Saudi nationals.

The most important findings of the study are (i) vegan prevalence 4.7% and vegetarianism 7.8%; (ii) vegan diet more prevalent in youth (77.8%) and females (79.6%); (iii) eating breakfast is common behavior of Saudi nationals (iv) eating behavior and physical activity level is more favorable among participants on vegetarian diet; and (v) consumption of fast-food and fizzy beverages are positively correlated with BMI.

The prevalence of vegetarian diet is much higher among Saudi nationals as compared to the vegetarian prevalence reported in Americans (5%) (7) and lower than Germans (10%) (11) and Indians (30%) (10). Moreover, the present results are similar to vegetarian prevalence among Canadians (8%) (8). However, differences in the social and cultural perspective between Saudi Arabia and other western nations may also affect the vegan/vegetarian diet adoption prevalence. Only one study is available from Saudi Arabia which reported a higher prevalence of veganism (8%) than vegetarianism (5%), contrary to our findings which might be due to the difference in the selection of the study population. Our study participants were only Saudi nationals, while study by AlHusseini et al. (26), included both Saudi nationals and residents (42 and 58%, respectively). Traditionally, the Saudi nationals follow a non-vegetarian diet, but changes in the perception of youth regarding nutrition, ethics, and health are reflected in increased adoption of vegan and vegetarian diet in the recent year. This change will lead to increased fiber intake and decreased consumption of unhealthy fat leading to positive impact on BMI (27).

Similar to our study results, a study from Germany also reported a significantly higher proportion female (*p* < 0.001) among vegetarians/vegans and were significantly younger than omnivores (*p* < 0.001) (28). These results are in line with most studies that show the same trend for more females being vegetarian than males, regardless of nation/culture (6–10, 21, 26). Moreover, there are studies that showed a strong association between red meat consumption and masculinity (29–32). Our study shows that participants on vegetarian diet have a lower BMI and a high physical activity level compared to non-vegetarian participants. These results are in accordance with other studies conducted in different parts of the world (7, 28). Moreover, the reason for adopting a vegetarian diet was to improve their health and control body weight, which supports the higher physical activity level in our study population. However, a study from Brazil reported no significant difference in the BMI and physical activity level between the vegetarian and omnivorous group (33). In our study, all underweight vegan participants and most of the vegetarian participants were young females. It is possible that these participants might have poorly planned their diet due to lack of knowledge about a healthy balanced diet. There are studies which have reported that a well-planned vegan/vegetarian diets can provide nutritional requirements to the individuals who are involved in endurance activities (14, 15, 21). The prevalence of underweight among vegan and vegetarian diet participants is high and need to be addressed through awareness programs on healthy balanced diet especially in colleges and universities as vegan and vegetarian diet adoption is higher among the youth. Moreover, students should be encouraged to consult dietitian when adopting vegan/vegetarian diet to achieve a healthy weight and to avoid micronutrient deficiency.

Our study results of eating behavior vary from a previous study, which reported a higher prevalence for eating breakfast (88.6%) at least three times per week, rarely eating vegetables and fruits (32.2 and 36.1%) and eating snacks (31.7%), while a lower prevalence 31.4% for eating three meals per day (34). This deviation in eating behavior may be due to the difference in study population which comprised of only 18–24 years male students. Further, there is a duration of more than 10-years between the two surveys, which might be another reason for a better eating behavior witnessed in our study. The eating behavior from nationally representative study of Saudi population revealed a low intake of vegetables and fruits, which are source of high-fiber content along with other nutrient and a higher consumption of protein and carbohydrate through meat products and rice (35, 36). An encouragement toward a well-planned vegan and vegetarian diet among Saudi population may help in controlling the obesity.

In the present study a significantly higher intake of vegetables and fruits was observed in participants on a vegetarian diet than on a non-vegetarian diet, while eating fast-foods and drinking fizzy drinks was more popular among participants on a non-vegetarian diet. Similar results have been reported in studies conducted in western countries (32, 37, 38). Our study findings indicate that the population following a vegan and vegetarian diet shows an overall healthier lifestyle with better eating behavior, and high level of physical activity than non-vegetarians. This finding is supported by a previous study conducted among the members of the Seventh-day Adventist Church which reports, irrespective of their religion-based lifestyles, that people following a vegetarian diet are usually less likely to smoke, drink less alcohol, and are more physically active (39).

There was significant decrease in the BMI of the participants who consumed more vegetable and fruit. However, a study from Saudi Arabia (34) reported no significant correlation between BMI and eating three meals a day and the consumption of vegetables and fruits, which may be due to the small sample size, and the age and gender of the participants. In our study, an increased consumption of fast food, canned food and fizzy drinks showed a significant association with body weight and subsequently BMI. Our study findings were similar to the results reported by other studies (40–42).

Though the present study provides important findings which were not reported earlier for Saudi nationals, the study has some limitations. The level of awareness of good eating behavior and importance of physical activity might have resulted in social desirability bias with higher responses to the good eating behavior and high level of physical activity. However, this study results are reliable firstly for prevalence of BMI categories as the BMI was calculated after taking the measurements and secondly for correlation of eating behavior with BMI as the correlation was performed by controlling for the confounder physical activity level.

## Conclusion

Our study shows that vegan and vegetarian diet have gained access into the lifestyle of Saudi adults with a prevalence of 4.7 and 7.8%, respectively, especially vegan diet was more prevalent amongst youth (77.8%) and females (79.6%). Participants on vegetarian diet showed better lifestyle like higher physical activity level, higher consumption of fruits, vegetables, dairy products and low intake of fast-foods and fizzy beverages.

## Data availability statement

The raw data supporting the conclusions of this article will be made available by the authors, without undue reservation.

## Ethics statement

The studies involving human participants were reviewed and approved by the Saudi Electronic University Research Ethics Committee. The patients/participants provided their written informed consent to participate in this study.

## Author contributions

MA-M made substantial contributions to conception and design, in charge of data collection and curation, writing of the manuscript, performed the statistical analysis, and approved the submitted version of the manuscript.

## Conflict of interest

The author declares that the research was conducted in the absence of any commercial or financial relationships that could be construed as a potential conflict of interest.

## Publisher's note

All claims expressed in this article are solely those of the authors and do not necessarily represent those of their affiliated organizations, or those of the publisher, the editors and the reviewers. Any product that may be evaluated in this article, or claim that may be made by its manufacturer, is not guaranteed or endorsed by the publisher.
